# Pathogenicity and virulence of *Rickettsia*

**DOI:** 10.1080/21505594.2022.2132047

**Published:** 2022-10-08

**Authors:** Luke Helminiak, Smruti Mishra, Hwan Keun Kim

**Affiliations:** Center for Infectious Diseases, Department of Microbiology and Immunology, Stony Brook University, Stony Brook, NY, USA

**Keywords:** Rickettsia, tick, spotted fever, rickettsiosis, pathogenesis, virulence

## Abstract

Rickettsiae include diverse Gram-negative microbial species that exhibit obligatory intracellular lifecycles between mammalian hosts and arthropod vectors. Human infections with arthropod-borne *Rickettsia* continue to cause significant morbidity and mortality as recent environmental changes foster the proliferation of arthropod vectors and increased exposure to humans. However, the technical difficulties in working with *Rickettsia* have delayed our progress in understanding the molecular mechanisms involved in rickettsial pathogenesis and disease transmission. Recent advances in developing genetic tools for *Rickettsia* have enabled investigators to identify virulence genes, uncover molecular functions, and characterize host responses to rickettsial determinants. Therefore, continued efforts to determine virulence genes and their biological functions will help us understand the underlying mechanisms associated with arthropod-borne rickettsioses.

## Rickettsioses: arthropod-borne diseases

Organisms belonging to the genus *Rickettsia* are obligate intracellular microorganisms with the ability to survive in both vertebrate and arthropod hosts. Rickettsiae are Gram-negative rod-shaped (0.3–0.5 × 0.8–1 µm) bacteria with genome sizes ranging between 1.1–1.5 Mbp (69–84% coding capacity) among different rickettsial species [1–3]. Rickettsiae have evolved to downsize their genome and purged many genes involved in various metabolic pathways. Thus, rickettsiae are incapable of living extracellularly and pilfer various nutrient components for their replication from the hosts [4]. Based on phylogeny, clinical symptoms, and antigenic properties, rickettsiae are classified into four groups: the spotted fever group (SFG), typhus group (TG), transitional group (TRG), and ancestral group (AG) [5–7]. The TG consists of two species, *R. typhi* (murine typhus) and *R. prowazekii* (epidemic typhus), that are transmitted by faeces of infected fleas and lice, respectively [8,9]. Members of the SFG include tick-transmitted pathogens such as *R. rickettsii* (Rocky Mountain spotted fever), *R. conorii* (Mediterranean spotted fever), and *R. parkeri* (mild-moderate spotted fever), among others [8,10,11]. With recent advances in molecular detection and identification, investigators continue to identify novel SFG *Rickettsia* species throughout the world [8,12]. The TRG includes the *R. felis* (flea-borne spotted fever), *R. australis* (tick-borne Queensland tick typhus), and *R. akari* (mite-borne rickettsialpox) [5,7,8]. Species belonging to AG, such as *R. bellii* and *R. canadensis*, are considered non-pathogenic and comparatively less studied than rickettsial species with proven pathogenicity in the other groups [5,6,8].

Humans are incidental hosts for *Rickettsia* and do not usually contribute to rickettsial transmission and maintenance. Instead, rickettsiae are maintained in mammalian hosts (e.g. small rodents) and/or their arthropod vectors (e.g. ticks and fleas). Rickettsioses occur when infected arthropod vectors feed on humans or contaminate skin openings or mucosal surfaces with *Rickettsia*-infected arthropod excrement. Unlike other *Rickettsia* species, humans serve as a reservoir for *R. prowazekii* [13]. The human body louse, *Pediculus humanus corporis*, is the principal vector but not a reservoir for *R. prowazekii* because infected lice die within a week as the pathogen replicates and damages the gut epithelium of the infected lice [13,14]. While the exact mechanisms are poorly understood, *R. prowazekii* can latently persist in patients and cause recrudescent Brill – Zinsser disease, potentially disseminating *R. prowazekii* months to years after the primary infection [15,16]. Most rickettsiae preferentially target vascular endothelial cells within the bloodstream, leading to local and systemic vascular injury and inflammation with tissue infiltration of leukocytes and thrombosis [17,18]. The clinical spectrum of rickettsial infections changes significantly from mild to severe diseases, with varying case-fatality rates [12,19,20]. Several factors, including age, prompt diagnosis and antibiotic treatment, underlying conditions, and infecting rickettsial agents, contribute to the variability in clinical outcomes [21]. Patients with rickettsioses often present non-specific clinical symptoms, including fever, rash, headache, malaise, and vomiting [21]. Some patients develop single or multiple eschars (tache noire, black spot) at the tick bite site, representing a dermal necrotic lesion associated with a primary inoculation site of *Rickettsia* and extensive rickettsial replication [22]. Patients also display maculopapular rashes as rickettsiae cause vascular damage and inflammation, leading to fluid escape into interstitial spaces [22]. As the rickettsial infections progress, patients appear delirious and exhibit neurological symptoms during the end stages of diseases [21]. While uncommon, extensive necrosis and gangrene of the extremities occur in severe cases of rickettsioses, requiring surgical interventions [21,23]. In the absence of timely intervention, infections with highly pathogenic *Rickettsia* species can cause life-threatening complications, such as severe vasculitis, encephalitis, sepsis, interstitial pneumonia with noncardiogenic pulmonary oedema and acute respiratory distress syndrome, and multiorgan failure [21,24–26].

During major wars, such as the first and second World Wars, poor hygiene conditions contributed to the onset of epidemic typhus, claiming millions of lives [27]. While the reported cases of epidemic typhus remain low nowadays, several environmental and circumstantial conditions (e.g. war, poverty, and natural disasters) associated with poor hygiene can trigger a new outbreak for *R. prowazekii*, as recently reported in Burundi (1997), Russia (1997–1998), Rwanda (2012), and Algeria (1998) [28–32]. Because of its potential to cause vector-transmittable severe illnesses, *R. prowazekii* has been weaponized by the Soviet Union [33]. On the other hand, flea-borne *Rickettsia* continues to cause local outbreaks in tropical and subtropical climates around the world, including the coastal states of California, Hawaii, and Southeast Texas [34–37]. In addition, recent environmental changes have promoted the expansion and invasion of several tick species with aggressive and non-specific biting behaviours, contributing to the rise of tick-borne rickettsioses in several parts of the world [19]. For all rickettsioses, prompt diagnosis and early antibiotic intervention are the keys to full recovery without complications [21]. However, untrained clinicians may have difficulty making differential diagnoses as non-specific febrile clinical presentations mimic other bacterial and viral infections. Molecular diagnostic tests (e.g. nucleic acid amplification) are available at reference diagnostic laboratories for accurate detection and identification of *Rickettsia* during the early phase of the disease [21,38]. However, these tests often yield variable results and are not readily available in endemic settings due to the high costs and specific skill sets required for analysis [39]. On the other hand, serological tests often identify the presence of non-specific and cross-reactive antibodies after the acute phase of the disease (7–10 days post-infection) and fail to reveal the causative agent [21,38]. The challenging nature of proper diagnosis for rickettsioses in the acute phase has promoted empirical antibiotic treatments without any subsequent confirmation, contributing to the delay in developing diagnostic tools and persistent underappreciation of clinical significance [40].

Due to their intrinsic nature to survive intracellularly and the lack of axenic growth media, rickettsiae have historically been recalcitrant to genetic manipulation. Over the past decades, several investigators have pioneered and made significant achievements in developing genetic tools for *Rickettsia*, identifying virulence factors, revealing the underlying molecular mechanisms, and understanding rickettsial biology and pathogenesis (Table 1). In this review, we discuss the pathogenesis of *Rickettsia* with a focus on virulence determinants of *Rickettsia*, their contributions to obligate intracellular lifecycles, their interactions with host cells, and the immune evasion strategies they utilize. We highlight these recent advances and how they have provided significant insights into understanding the unique intracellular lifecycle of *Rickettsia*.

**Table 1.** List of putative rickettsial virulence genes.

Gene	Protein	Putative functions	Predicted secretion pathways
*sca0*	Outer membrane protein A	Interacts with FGFR1 and α2β1 integrin and aids in the attachment and invasion to host cells	Sec-T5SS
*sca1*	Surface cell antigen 1	Involved in the attachment to host cells	Sec-T5SS
*sca2*	Surface cell antigen 1	Formin-like activity, nucleates actin and helps in the cell-to-cell spread at later stages of infection	Sec-T5SS
*sca4*	Surface cell antigen 1	Inhibits interaction of vinculin – α catenin at the focal adhesion sites and disturbs adherent junction complexes to aid in cell-to-cell spread	Sec-unknown
*sca5*	Outer membrane protein B	Interacts with Ku70 on the host cell surface and participates in the attachment and invasion to host cells. Blocks ubiquitination of OmpA and prevents autophagy	Sec-T5SS
*tlyA*	Haemolysin A	Assists rickettsial escape from vacuolar structures	Unknown
*tlyC*	Haemolysin C	Assists rickettsial escape from vacuolar structures	Unknown
*pat1*	Patatin-like phospholipase 1	Phospholipase activities involved in vacuole escape and avoidance of autophagy killing	Unknown
*pat2*	Patatin-like phospholipase 2	Phospholipase activities involved in vacuole escape	Unknown
*pld*	Phospholipase D	Phospholipase activities involved in vacuole escape	Unknown
*rarp1*	Rickettsial ankyrin repeat protein 1	Unknown	Sec-TolC
*rarp2*	Rickettsial ankyrin repeat protein 2	Cysteine protease activities involved in dispersing trans-Golgi network	T4SS
*rickA*	Arp2/3 complex-activating protein	Mimics WASP and activates Arp2/3 to form actin tails and mediate motility at the early stages of infection	Unknown
*ralF*	Guanine nucleotide exchange factor	Expressed early in infection activates Arf6 and mediates invasion	T4SS
*risk1*	Rickettsia intracellular secreted kinase-1	Phosphatidylinositol 3-kinase, which contributes to intracellular growth by directly engaging the Beclin-1 complex, facilitates internalization and escape into the host cytosol.	T4SS

## Rickettsial intracellular lifecycle and virulence determinants

**Figure 1.**
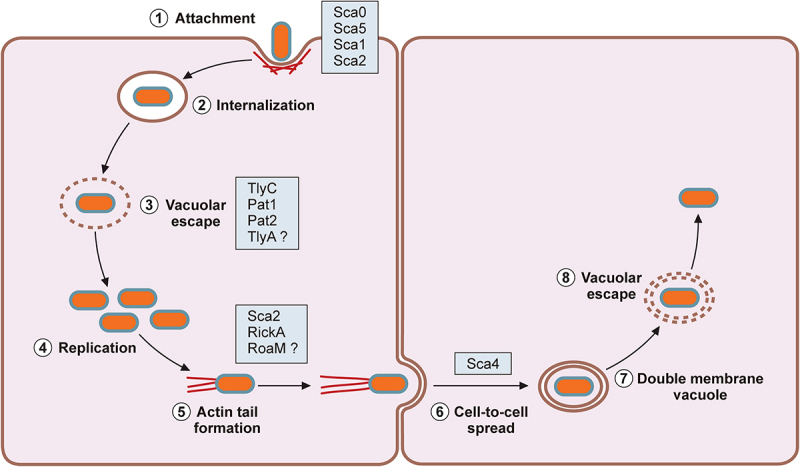
Rickettsial intracellular lifecycle.

In mammalian hosts, pathogenic *Rickettsia* species target vascular endothelial cells and infiltrating immune cells, induce actin-mediated endocytosis, and escape into the cytoplasmic compartment (Figure 1). Pathogenic *Rickettsia* species have evolved mechanisms to avoid intracellular detection and elimination of pathogens within the host cells. As a result, infections with pathogenic *Rickettsia* induce increased vascular permeability associated with rickettsial replication and disruption of vascular endothelial cells with perivascular infiltration of T cells and macrophages [41]. Progressive endothelial cell injury leads to the generation of characteristic erythematous rash, disseminated vasculitis, cutaneous necrosis, pneumonitis, meningoencephalitis, and multiorgan failure [13,21,39,42]. On the other hand, previous investigations show that infection of vascular endothelial cells with *Rickettsia* activates a proinflammatory state and induces cytokine and chemokine responses [43–47]. Thus, the molecular interactions between *Rickettsia* and endothelial cells have a significant role in rickettsioses. As rickettsiae continue to replicate and spread through the vasculature, perivascular neutrophilic and lymphohistiocytic inflammatory cells infiltrate into the site of infection to prevent further dissemination of the invading bacteria [48,49]. Recent investigations demonstrated that pathogenic *Rickettsia* species, such as *R. rickettsii* and *R. conorii*, have evolved to resist bactericidal mechanisms and establish a replicative niche within the cytosolic compartments of professional phagocytes, such as macrophages, suggesting that rickettsial survival in immune cells may contribute to rickettsial virulence and pathogenesis [50,51]. For instance, *R. conorii* replication in THP-1 macrophages induced unique proteome signatures and altered metabolic and lipid catabolic pathways, favouring anti-inflammatory M2 responses [52,53]. Here, we highlight recent investigations on the pathogenic mechanisms enabling *Rickettsia* to exploit and evade host immune protection mechanisms and establish an intracellular niche for their survival and transmission within mammalian hosts.

1. *Rickettsia* attaches to host cells via interaction of rickettsial surface proteins with host cell receptors; 2. Downstream signaling cascades lead to the internalization of *Rickettsia*; 3. Using several membranolytic factors, *Rickettsia* escapes the endocytic vacuoles; 4. *Rickettsia* resides within the host cytosol and replicates while taking nutrients from the host; 5. Some *Rickettsia* species exhibit actin-based motility; 6. *Rickettsia* undergoes cell-to-cell spread; 7. *Rickettsia* breaks double vacuolar membrane structures; 8. *Rickettsia* resides within the cytosolic compartment and reinitiates the infectious cycle.

### Secretion Systems in Rickettsia

**Figure 2.**
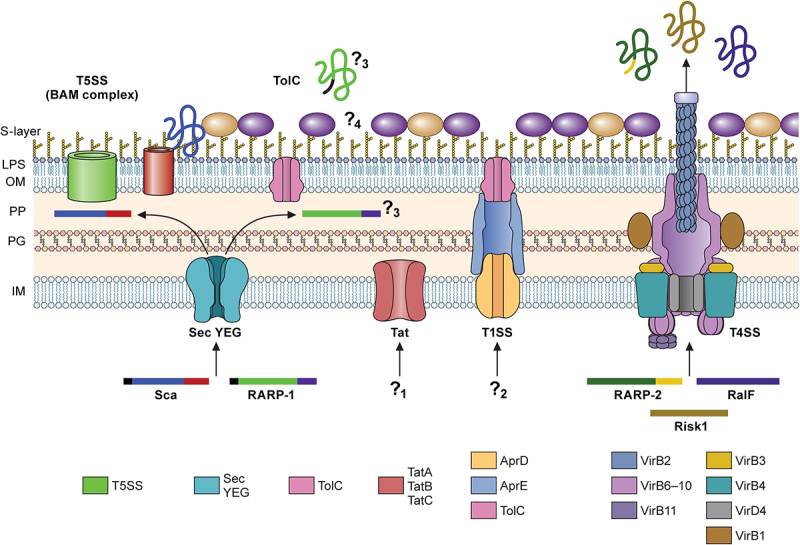
Secretion systems in *Rickettsia*.

Bacterial surface and secreted proteins are responsible for numerous virulence mechanisms, such as sensing the environmental changes, protecting the microorganisms from stresses, adhering to and invading host cells, neutralizing innate immune components, and combating professional phagocytes. Similarly, *Rickettsia* utilizes diverse sets of surface-exposed proteins or secreted proteins to orchestrate its obligate intracellular lifecycles within target cells and establish a successful infection. Genome analyses identified five potential secretion systems in *Rickettsia*: Sec translocon, twin-arginine translocation pathway, Type I secretion system (T1SS), Type IV secretion system (T4SS), and Type V secretion system (T5SS) (Figure 2) [54]. These analyses identified the presence of functional Sec translocon components in all *Rickettsia* species. Interestingly, rickettsiae lack two genes encoding LolE and LolB involved in the localization of the lipoprotein (Lol) pathway for the secretion of lipoproteins to the outer membrane [54]. It is predicted that homodimer pairs of LolC and LolD make a functional unit of Lol transporters in *Rickettsia*. Several lines of experimental evidence suggest that *Rickettsia* employs a functional Sec secretion system [54]. In *R. rickettsii*, RT-PCR analysis identified the presence of monocistronic transcript for *secA* gene [55]. Further, heterologous expression of *lepB* from *R. rickettsii* and *R. typhi* and *lspA* from *R. typhi* complemented *E. coli* variants and confirmed signal peptidase activities [56,57]. While *in silico* secretion signal prediction tools successfully identified several Sec substrates in *Rickettsia*, recent studies suggest the presence of noncanonical secretion peptide sequences in *Rickettsiales* [58].

*Rickettsia* utilizes five secretion pathways: Sec-T5SS, Sec-TolC, Tat, T1SS, and T4SS. Most Sca proteins have three domains: the N-terminal secretion signal (black), passenger domain (blue), and C-terminal autotransporter domain (red). The black and purple boxes on RARP-1 denote the N-terminal secretion signal and C-terminal Ankyrin repeat domain. The C-terminal Ankyrin repeat domain of RARP-2 is colored in yellow. Question marks 1 and 2 indicate uncharacterized proteins that travel through the Tat and/or T1SS. Question mark 3 indicates divergent destinations of RARP-1 in *Rickettsia*. Lastly, question mark 4 indicates unresolved compositions of S-layer proteins and their interactions with the outer membrane components, such as O-Ag. IM-inner membrane; PP-periplasm; PG-peptidoglycan; OM-outer membrane; LPS-lipopolysaccharide; S-layer-surface layer.

*Rickettsia* encodes seventeen outer membrane proteins (Omp), also known as surface cell antigens (Sca), which contain an *N*-terminal secretion signal for Sec-dependent secretion to the periplasmic space, followed by a passenger domain for virulence function, and a C-terminal β-barrel domain for protein translocation to outer-membrane via Type V secretion pathway (T5SS) [54,59–61]. Thus, the current model for the *Rickettsia* Sca secretion suggests that the Sec secretion machinery engages the *N*-terminal secretion signal of Sca proteins and translocates the polypeptides into the periplasmic space. Within the periplasmic area, the chaperone Skp and rickettsial β-barrel assembly machinery (BAM) assist the insertion of the C-terminal β-barrel domain in the outer membrane [54,62]. Then, conformational changes in the β-barrel domain facilitate the secretion of the passenger domain and dissociation from the BAM complex. The passenger domains of the Sca proteins are cleaved from the β-barrel domain by uncharacterized surface-associated enzymes or autocleavage reactions but remain non-covalently associated with the β-barrel domain on the surface of rickettsial cells [63,64]. It is interesting to postulate that the dissociated β-barrel domains may facilitate the translocation of Sca proteins lacking dedicated β-barrel domains or serve as outer-membrane channels for other molecules. On the other hand, the turnover rate of the β-barrel domains may dictate the release of the passenger domain to the extracellular spaces and facilitate the proper display of surface proteins to different environmental stimuli. However, the underlying molecular interactions between the passenger and β-barrel domains remain largely unresolved. As outer membrane proteins, Sca polypeptides constantly interact with host cytoplasmic or organelle proteins and are shown to be immunogenic, making them ideal candidates for understanding rickettsial biology and pathogenesis and developing vaccine antigens (see *Attachment and Invasion*).

TolC forms an outer-membrane channel in Gram-negative bacteria and functions as the exit tunnel for the Type I secretion system (T1SS) and drug efflux conduit associated with multidrug resistance. Thus, TolC plays a critical role in the virulence of Gram-negative bacterial pathogens. Current bioinformatics and experimental evidence support the conservation and presence of TolC in the outer membrane of *Rickettsia* [5,54,65]. However, little is known about the molecular functions and TolC substrates in *Rickettsia*. A previous study suggested that *Rickettsia* ankyrin repeat protein 1 (RARP-1) is secreted and deposited in the cytosolic compartment of *R. typhi*-infected mammalian (Vero76 and HeLa) cells [65]. Further analyses using a surrogate *E. coli* expression system determined that the secretion of recombinant RARP-1 is dependent on the Sec-TolC pathway [65]. Interestingly, recent work demonstrated that, in contrast to *R. typhi* RARP-1, *R. parkeri* RARP-1 is not detected in the host cytoplasmic compartment [66]. Rather, *R. parkeri* RARP-1 is secreted to the periplasmic space and interacts with other rickettsial factors that may mediate the secretion of other molecules or contribute to bacterial physiology [66]. Ankyrin repeat proteins in *Pseudomonas aeruginosa* and *Bdellovibrio bacteriovorus* also reside within the periplasm and play various roles in maintaining bacterial physiology [67,68]. Given that RARP-1 is conserved across the genus, continued studies are necessary to determine the interacting partners in the periplasmic area and biological roles in the rickettsial physiology and virulence. It is also interesting to postulate that additional factors are involved in the secretion of RARP-1 in *Rickettsia*, contributing to different secretion profiles in *R. typhi* and *R. parkeri*.

The twin-arginine translocation (Tat) pathway in Gram-negative microorganisms is responsible for the secretion of fully folded substrates, including virulence factors, from the cytoplasmic area to the periplasmic space. Tat substrates are unique in that the *N*-terminal secretion signals contain a consensus “twin-arginine” motif at the distal end of the basic region [69,70]. Previous *in silico* analyses identified Tat genes (*tatA*, *tatB*, and *tatC*) dispersed throughout all rickettsial genomes but failed to reveal Tat substrates in *Rickettsia*, except PetA with a putative Tat signal peptide [54]. Similar to the sequence variations in Sec secretion substrates, Tat substrates may vary substantially in *Rickettsia*, making it difficult to predict by the algorithms optimized for other bacterial organisms. Thus far, no substrates have been experimentally tested in *Rickettsia*. Future research is essential to determine the functionality of the Tat system and targeted substrates for their roles in rickettsial intracellular pathogenesis.

The T1SS is most well-characterized for a pore-forming toxin, haemolysin A (HlyA), in *E. coli*, illustrating the conserved and essential features of the T1SS. Additional cargos for T1SS include adhesins, iron-scavenger proteins, lipases, and proteases, with their sizes ranging from 20 kDa to 1,500 kDa, suggesting their diverse roles in bacterial pathogenesis [71]. While the mechanism by which cargo molecules interact with the T1SS is ill-defined, the T1SS substrates are often defined by the presence of a GG repeat motif in the C-terminal end with varying degrees of conservation. In *Rickettsia*, the bioinformatic analysis identified a conserved operon encoding homolog proteins (e.g. Rc0427 – Rc0429 in *R. conorii*) capable of assembling into a functional T1SS: an inner membrane ATP binding cassette transporter, a periplasmic membrane fusion protein, and a multifunctional outer membrane protein of the TolC family (located in a separate location) [54]. Unlike *E. coli hly* operon, *Rickettsia* does not have a gene encoding a HlyC homolog, which catalyzes post-translational acylation on two internal lysine residues of HlyA. Thus far, no known T1SS substrates have been characterized for *Rickettsia*. However, as recent studies characterized T1SS effector proteins in *Ehrlichia* and *Orientia*, it is highly probable that *Rickettsia* utilizes T1SS and TolC for the delivery of effector molecules that contribute to rickettsial pathogenesis and arthropod colonization and transmission. Additional studies are essential to characterize the T1SS substrate motif in *Rickettsia* and the underlying secretion mechanisms for T1SS.

The Type IV secretion system (T4SS) forms large protein complexes that traverse the cell envelope of bacterial organisms and deliver various molecules to bacterial or eukaryotic target cells, specifically for bacterial conjugation and secretion of effector molecules. The T4SS is the most versatile and divergent secretion system divided into subgroups based on genetic organization and evolution. For instance, the initial characterization of the incompatibility group of the representative conjugative plasmids subgrouped the T4SS into three major types: IncF, IncP, and IncI. Alternatively, T4SS that displays similarities to the *Agrobacterium tumefaciens* VirB/D4 system (IncF and IncP, comprised of 12 core proteins, VirB1 – VirB11 and VirD4) was designated as a Type IVA system, whereas genetic determinants closely related to the archetypal *dot*/*icm* (defective in organelle trafficking/intracellular multiplication) system of *Legionella pneumophila* was categorized as Type IVB system [72]. The third group is comprised of those that display minimal homologies to IVA and IVB systems. Previous bioinformatic analyses suggest that *Rickettsia* encodes atypical *P*-T4SS (T4ASS), named as rickettsiales *vir* (virulence) homologs (*rvh*), with unprecedented gene duplication (*rvhB9*, *rvhB8*, and *rvhB4*) and proliferation (3–5 copies of *rvhB6*) dispersed throughout the genome [54,73]. Interestingly, the *rvh* T4SS encodes all known VirB/D4 components except for the VirB5, a minor component of the extracellular T pilis, corroborating current experimental evidence that *Rickettsia* lacks pili-like structures on the cell surface (with the exception of *R. bellii* and *R. felis*, potentially due to the presence of incomplete *tra* genes) [54,74,75]. It is currently unknown how the anomalous *rvh* system assembles the secretion apparatus through which diverse substrate molecules translocate into host cells. The secretion of effector proteins and their biological roles during infection have been described for T4SS in *Ehrlichia* and *Anaplasma* [76,77]. For *Rickettsia*, a recent bacterial two-hybrid analysis demonstrated that RalF interacts with RvhD4, a homolog of VirD4 that recognizes target molecules and regulates substrate translocation in other *P*-T4SS, suggesting *rvh*-mediated secretion of RalF [78]. The *dot*/*icm* T4BSS is known to secrete RalF comprised of an N-terminal Sec7 domain, which functions as a guanine nucleotide exchange factor of ADP-ribosylation factors (Arf), and a C-terminal Sec7-capping domain that regulates active site access to Arf [79]. The *L. pneumophila* RalF recruits host Arf1 to the *Legionella*-containing vacuole (LCV) and facilitates the endoplasmic reticulum membrane fusion to LCV [80]. Interestingly, RalF is pseudogenized in all SFG *Rickettsia* but present in TG, TRG, and AG *Rickettsia* with variable lengths of uncharacterized C-terminal domain with a conserved Pro-rich region and a *rvh* secretion signal. Experiments with *in vitro* tissue culture infections and ectopic expression analysis of RalF variants demonstrated that RalF is expressed early during host cell infection and modulates *R. typhi* invasion by activating Arf6, which subsequently recruits phosphatidylinositol 4-phosphate 5-kinase to the site of entry, enriching PI(4,5)P_2_ on the plasma membrane, and leading to host cytoskeleton remodelling [81]. Another ankyrin repeat protein, RARP2, is recently shown to interact with RvhD4 and is predicted to be secreted by *rvh* T4SS [82,83]. RARP2 consists of a highly conserved N-terminal domain with putative cysteine protease activity, followed by a variable number of ankyrin repeats and a distinct C-terminal tail [83]. Comparative genomic analysis suggests that the increasing number of ankyrin repeats is positively associated with rickettsial virulence and clear plaque formation in *Rickettsia*-infected tissue culture cells [83]. During host cell infections, RARP2 is expressed early and shown to be deposited in the endoplasmic reticulum [83]. However, the host cellular target for RARP2 is unknown. This work demonstrated that the cysteine protease activity and proper ankyrin repeats are necessary to disperse the *trans*-Golgi network by unknown mechanisms, potentially perturbing protein trafficking and sorting [82]. Similar to other viral and bacterial infections, inhibiting protein trafficking to the plasma membrane may contribute to rickettsial pathogenesis and immune evasions by disrupting cellular barrier functions and downregulating immune recognition molecules, such as MHC-I [82]. Additional genetic and functional studies are necessary to identify additional *rvh* substrates, determine their biological roles during the rickettsial obligate intracellular lifecycle, and define molecular functions of *rvh* components in forming the T4SS apparatus.

### Attachment and invasion

**Figure 3.**
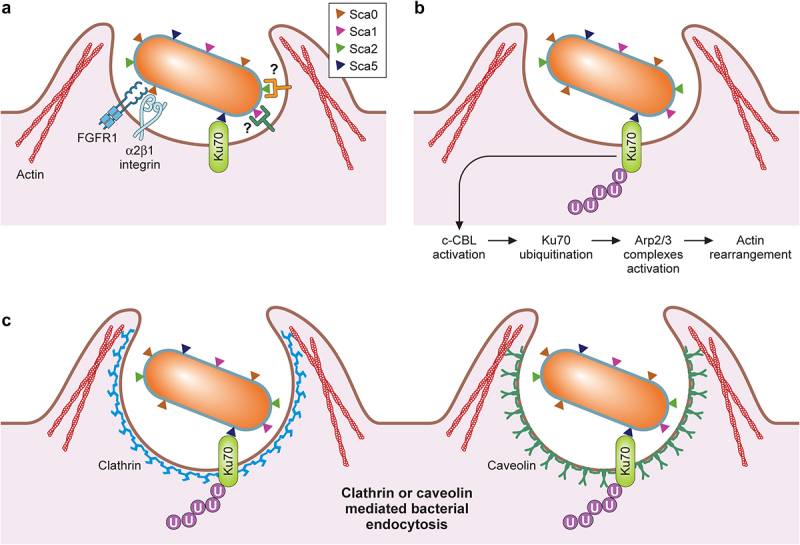
Rickettsial determinants in host cell attachment and internalization.

In the absence of metabolic genes, rickettsiae must invade the host cytoplasm and acquire nutrients for survival and replication. Thus, rickettsial adherence and entrance into host cells are critical steps in initiating rickettsioses. Intracellular bacterial organisms utilize two general strategies, “zipper” or “trigger”-mechanisms, to enter host target cells [1,84]. The “zipper”-mechanism requires bacterial surface proteins, such as adhesins and invasins, to make direct physical contact with host receptors, initiating downstream signalling cascades for cytoskeletal rearrangement and bacterial internalization, as reported for *Yersinia* and *Listeria* species [84]. On the other hand, the “trigger”-mechanism involves the injection of effector proteins by Type III and Type IV secretion systems and remodelling of host factors for the bacterial uptake, as described for *Salmonella* and *Shigella* species [84]. While the *rvh* T4SS is present in all species of *Rickettsia*, additional studies are required to determine the role of *rvh* T4SS during the host cell invasion of *Rickettsia* (see *Secretion Systems*). Thus, it remains unknown whether *Rickettsia* utilizes the trigger mechanism for invasion. On the other hand, rickettsial surface proteins interact with host receptors and induce actin-mediated endocytosis (Figure 3). Transmission electron microscopic analyses of non-phagocytic Vero cells interacting with *R. conorii* suggest that rickettsial entry is similar to *L. monocytogenes* and employs a “zipper”-mechanism also known as induced phagocytosis [85–87].

A. Rickettsial entry into mammalian cells is mediated by interactions of its surface antigens- Sca0 with α2β1 integrin and FGFR1 receptor, Sca5 with Ku70, and Sca1 and Sca2 with unknown receptors on the cell surface; B. Ku70-Sca5 interaction leads to activation of E3 ubiquitin ligase c-Cbl causing ubiquitination of Ku70. This initiates several downstream host-signaling pathways leading to activation of the Arp2/3 complexes resulting in actin rearrangement; C. This induces bacterial internalization via actin, clathrin, and caveolin 2- mediated endocytosis.

Sca5, also known as rickettsial outer membrane protein B (rOmpB), is conserved in all *Rickettsia* species [59]. Sca5 is secreted via the Sec-T5SS pathway, abundantly expressed on the rickettsial surface, and predicted to form a paracrystalline surface layer (S-layer) [54,59,88–90]. Previous biochemical studies identified Ku70 as a host receptor for Sca5 [91]. Ku70 is predominantly expressed in the nucleus of most cells, forming a heterodimer with Ku80, which plays an essential role in the non-homologous end-joining pathway for DNA double-strand break repairs [92]. In addition, Ku70 is also involved in cytosolic DNA-sensing and immune signalling pathways in various cell types [93]. Interestingly, Ku70 has also been found in cholesterol-rich lipid rafts on the plasma membrane and facilitates interactions with metalloprotease-9 and fibronectin [94,95]. Following Sca5 interaction with Ku70, downstream signalling pathways activate Arp2/3 complexes, rearranging host actin filaments around invading *Rickettsia*, and induce clathrin- and caveolin-mediated endocytic pathways [96,97]. However, it remains unknown whether Sca5 interactions with Ku70 result in diminished levels of Ku70 and downregulation of cytoplasmic Ku70 activities for rickettsial immune evasion. The expression of recombinant Sca5 in *E. coli* was sufficient to increase the level of bacterial attachment and invasion into non-phagocytic tissue culture cells [97–99]. Also, siRNA-based depletion of Ku70 and the pretreatment with Ku70-specific antibodies reduced *R. conorii* entry into Vero cells [91,97]. Both studies corroborate that Sca5 interactions with Ku70 mediate rickettsial uptake by host cells. Recent genetic studies also suggest that Sca5 facilitates rickettsial invasion as *R. parkeri sca5* mutant displayed slower invasion kinetics than wild-type *R. parkeri* infecting HMEC (human microvascular endothelial cells) cells (30 minutes vs. 20 minutes for *sca5* vs. wild-type) [100]. Interestingly, the *R. parkeri sca5* mutant ultimately reached the same invasion level in later time points, suggesting that additional rickettsial factors contribute to the attachment and invasion.

Sca0, also known as rOmpA, is another putative adhesin highly conserved in the SFG *Rickettsia*. It is currently postulated that Sca0, together with Sca5, constitute the rickettsial S-layer, but the exact molecular interactions between Sca0 and Sca5 for S-layer formations on *Rickettsia* are poorly understood [101]. Initial work indicated that Sca0-specific monoclonal antibodies and purified Sca0 perturbed the *R. rickettsii* interactions with mouse fibroblast L929 cells [102]. Furthermore, when expressed in *E. coli*, *R. conorii* Sca0 on the *E. coli* cellular surface increased *E. coli* attachment to and invasion into HeLa (human epithelial cells) and HMVEC-L (human lung microvascular endothelial cells) at a comparable level to *E. coli* expressing Sca5 [103]. Affinity chromatography analysis with a truncated recombinant Sca0 (A.A. 954–1735) identified α2β1 integrin as a putative host receptor for Sca0 [103]. Of note, the bioinformatic analysis identified an atypical conserved RNIGD motif within the recombinant Sca0, potentially involved in direct interaction with α2β1 integrin [103]. Additional studies with α2β1 integrin-specific monoclonal antibodies and siRNA knockdown experiments in HMVEC-L have verified that Sca0 interacts with α2β1 integrin and mediates rickettsial attachment and invasion into endothelial cells [103]. On the other hand, a recent study illustrated a potential interaction of the Sca0 β-barrel domain with fibroblast growth factor receptor-1 (FGFR1) and subsequent activation of FGFR1, which promoted caveolin-1-dependent uptake of *R. rickettsii* and *R. conorii* by endothelial cells [104]. Inhibition of FGFR1 with a small molecule inhibitor (AZD4547) resulted in a substantial decrease in rickettsial burden in tissue culture endothelial cells and the lungs of *R. conorii*-infected C3H/HeN mice [104]. In addition, siRNA knockdown of FGFR1, but not FGFR2, reduced the abundance of *Rickettsia* in endothelial cells, corroborating an important role for FGFR1-Sca0 interactions in the rickettsial invasion of endothelial cells [104]. Interestingly, infections of endothelial cells with *R. conorii* significantly reduced the abundance of microRNA (miRNA)-424 and miRNA-503, leading to increased mRNA levels of FGFR1 and fibroblast growth factor 2 (FGF2), a primary ligand for FGFR1 [105]. While it isn’t entirely clear how the increased levels of FGFR1 and FGF2 impact rickettsial pathogenesis, one can postulate that activated FGFR1 may facilitate the proliferation and prolonged survival of endothelial cells for *Rickettsia*. However, it remains unknown how the Sca0 β-barrel domain, which sits below the S-layer as an integral membrane protein, avoids steric hindrance and successfully interacts with FGFR1. It is possible that the interactions of the passenger domains of Sca0 and Sca5 with their respective host receptors induce conformational changes and allow the β-barrel domains to bind FGFR1 and other uncharacterized host receptors. Thus, continued studies are required to uncover molecular mechanisms by which *Rickettsia* activates FGFR1 and controls the miRNA levels in infected endothelial cells. It is important to note that another study addressed the role of Sca0 in rickettsial pathogenesis by generating a *sca0* knockout by insertion of a premature stop codon in *R. rickettsii* using a group II intron system [106]. In their study, *Noriea et al*. demonstrated that *R. rickettsii sca0* mutant did not show any attachment or growth defects in Vero cells and displayed a similar capacity to cause spotted fever disease in guinea pigs compared to the wild-type *R. rickettsii* [106]. Similarly, *R. parkeri* Sca0 was not required for invading endothelial cells [100]. This data strongly argues that *Rickettsia* utilizes multiple redundant pathways to ensure their successful invasion into the nutrient-rich host cytoplasm (Figure 3).

Other surface-exposed rickettsial proteins have been proposed to interact with host cells and facilitate rickettsial attachment and entry into the cytosolic compartment. For instance, *E. coli* expressing Sca1, a conserved polypeptide present in all *Rickettsia* species except for *R. prowazekii* and *R. canadensis*, enhanced *E. coli* attachment to non-phagocytic tissue culture cells but failed to facilitate *E. coli* invasion into the cytosol [107,108]. A similar experimental approach also determined that *E. coli* expressing Sca2, which is conserved in most SFG *Rickettsia*, displayed increased levels of attachment and invasion into multiple tissue culture cells [109]. Corroborating these studies, preincubations of tissue culture cells with soluble recombinant passenger domains of Sca1 and Sca2 perturbed the interactions of *R. conorii* or *E. coli* expressing Sca2 with host cells, respectively [108,109]. However, *R. rickettsii* and *R. parkeri* without *sca2* did not show significant defects in host cell invasion or growth [110,111]. Thus, additional genetic, biochemical, and molecular studies are necessary to determine the specific host receptors, identify involved protein domains, and reveal the subsequent molecular events facilitating rickettsial attachment and invasion into host cells. Recent advances in genetic analysis of *Rickettsia* permit investigators to study the biological roles of surface-exposed polypeptides, including conserved Sca proteins in rickettsial attachment and invasion into host cells, an essential step for rickettsial pathogenesis. Further, as demonstrated by *Engström et al*.,2019 future studies need to analyse the attachment and invasion kinetics to dissect multiple molecular mechanisms dedicated to the rickettsial invasion of host cells [100].

#### Membranolytic factors of Rickettsia

Intracellular pathogens are primarily divided into two groups, depending on their survival strategies. The first group of intracellular pathogens have evolved diverse mechanisms to survive and replicate within vacuolar structures and subvert host cellular immunity pathways by actively modifying endocytic vacuoles and modulating host-derived membrane and nutrient trafficking. On the other hand, the second group of intracellular pathogens actively escape vacuolar structures and proliferate in nutrient-rich cytosol. Microscopic analyses of *Rickettsia*-infected cells suggest that *Rickettsia* readily exits endocytic vacuoles and resides in the cytosolic compartment. Previous bioinformatic analyses identified two different classes of rickettsial determinants that may allow rickettsial escape from endosomes: phospholipases (*pat1*, *pat2*, and *pld*) and haemolysins (*tlyA* and *tlyC*) [54].

Many bacterial pathogens produce diverse sets of phospholipases to hydrolyse glycerophospholipids and modulate host cell membrane dynamics, signalling pathways, and cellular physiology. Previous work documented various molecular mechanisms of bacterial phospholipases involved in multiple host cellular processes. *Rickettsia* produces two patatin (Pat)-like proteins with phospholipase A_2_ (PLA_2_) activities (Pat1 and Pat2) and a phospholipase D (Pld) [112–114]. Three genes responsible for rickettsial phospholipases share low sequence homologies and are found in different genomic locations. PLA_2_ enzymes catalyse the hydrolysis of the sn-2 acyl bond of membrane phospholipids, releasing free fatty acid and lysoglycero-phospholipids [115]. On the other hand, Pld catalyzes the hydrolysis of the phosphodiester bond of glycerophospholipids to separate the head group from phosphatidic acid. Comparative genome sequence analysis determined that *pat1* is present in all *Rickettsia*, but *pat2* is often pseudogenized in many rickettsial species in the SFG, suggesting an ongoing reductive genome evolution process surrounding the *pat2* gene [114]. The vital role of PLA_2_ activities in rickettsial infections of tissue culture cells has been documented for various rickettsial pathogens, including *R. typhi*, *R. prowazekii*, *R. rickettsii*, and *R. conorii* [116–119]. For instance, during Vero cell infections, *R. typhi* produced Pat1 and Pat2, deposited them on the bacterial surface, and secreted polypeptides into host cell cytosol by uncharacterized secretion pathways. Antibodies targeting Pat1 and Pat2 recognized the surface-associated molecules and delayed rickettsial escape from phagolysosome, preventing *R. typhi* exit into the cytosol of Vero cells [114]. Interestingly, in the presence of host cell lysates, PLA_2_ activities of Pat1 and Pat2 increased significantly, implicating the existence of uncharacterized host cofactors altering the enzymatic kinetics [114]. By characterizing *R. parkeri pat1* mutant, a recent study confirmed that Pat1 plays a crucial role in facilitating *R. parkeri* escape from primary and secondary vacuoles and *R. parkeri* avoidance of autophagy killing [120]. Interestingly, *R. parkeri pat1* mutant did not show any growth defects in HMEC cells but generated smaller plaques than wild-type *R. parkeri* with diminished virulence in *Ifnar1*^−/−^ *Ifngr1*^−/−^ double knockout mice [120].

Similar to *pat1*, *pld* is conserved in *Rickettsia*. The biological role of *Rickettsia* Pld was first described in a surrogate microorganism, *Salmonella enterica* serovar Typhimurium, a facultative intracellular bacterial organism that establishes replicative niche in *Salmonella*-containing vacuole (SCV) in host cells [121]. Interestingly, introducing an expression vector that permits *R. prowazekii pld* expression under the *R. prowazekii* endogenous promoter in *Salmonella* allowed the pathogen to escape the SCV and relocate to the cytosolic compartment [121]. Further, pretreatments of *R. conorii* and *R. prowazekii* with affinity-purified α-Pld mouse polyclonal antibodies reduced Vero cell cytotoxicity measured after seven days of infection [113]. These results suggest an important role for Pld in rickettsial invasion into the cytosolic compartment. However, *R. prowazekii pld* mutant infecting mouse macrophage cells, RAW264.7, did not show defects in their escape into the cytosol and intracellular growth [122]. It is undetermined whether the remaining phospholipases and haemolysins (described below) compensated for the loss of *pld* in *R. prowazekii* in macrophages. Regardless, *R. prowazekii pld* mutant failed to cause clinical diseases over the 14 days of infection in guinea pigs, suggesting that Pld is a putative virulence factor for rickettsioses [122]. These studies demonstrate how important it is for *Rickettsia* to escape from the maturing endosomes and replicate in the cytoplasmic compartment. However, it is equally important for *Rickettsia* to remain in the cytosol and avoid host immune surveillance systems without breaking the integrity of the cellular membrane structures. Thus, future studies must reveal how *Rickettsia* regulates the cytotoxic activities of phospholipases, which may act as a double-edged sword for the rickettsial intracellular lifecycle [112]. Additional experiments are also necessary to determine the biological roles of lipid byproducts generated by rickettsial phospholipases in regulating host immune signalling and vascular inflammation.

Pore-forming haemolysins serve as virulence factors for diverse human pathogens, including *Escherichia coli*, *Listeria monocytogenes*, and *Staphylococcus aureus* [123–125]. The haemolytic activity of TG *Rickettsia* has been documented as early as 1948 by Clarke and Fox [126]. Since then, the haemolytic principles of TG *Rickettsia* have been examined in various experimental conditions. These studies suggest that 1) TG *Rickettsia* presents slow haemolytic kinetics in lysing erythrocytes of multiple mammalian species, 2) metabolically inactive *Rickettsia* fails to cause haemolysis, and 3) *Rickettsia* may cause contact-dependent haemolysis [127–129]. Recent whole-genome sequencing analyses revealed that *Rickettsia* has two conserved genes, *tlyA* and *tlyC*, encoding putative haemolysins. Introducing an expression vector harbouring *R. typhi tlyC* into two non-haemolytic microorganisms, *E. coli* and *Proteus mirabilis*, allowed the bacteria to confer haemolytic phenotypes with sheep erythrocytes [130]. Similar to the experiment described for Pld, the expression of TlyC in *Salmonella* allowed the pathogen to escape the SCV and reside in the cytosolic compartment [121]. However, the relative abundance of the bacteria in the cytosol was much higher for *Salmonella* expressing Pld [121]. The molecular mechanisms associated with haemolysin secretion and haemolysis remain poorly defined. Plus, the significance of haemolysis for rickettsial pathogenesis is not well understood. Lastly, additional studies need to address whether rickettsial haemolysins provide synergistic activities with rickettsial phospholipases in altering host cellular membrane structures for the rickettsial intracellular lifecycle.

#### Intracellular immune evasion

Initially characterized as a survival strategy for nutrient-deficient cells, autophagy is a highly conserved cellular immune strategy to target and engulf invading microorganisms in double-membrane vesicles and subsequently eliminate them by fusing with lysosomes. For successful infections, intracellular pathogens have developed diverse immune escape strategies and mechanisms to avoid or exploit autophagosomes to establish intracellular niches for replication. Recent studies highlight how different *Rickettsia* species have evolved to interact with these complex processes for their survival and pathogenesis. For example, *R. parkeri* employs two protein-lysine methyltransferases (PKMT1 and PKMT2) that modify the surface proteins and prevent polyubiquitylations [131]. Of the two enzymes, PKMT1 plays a significant role in the methylation of surface antigens, including Sca5. In the absence of PKMT1 or Sca5, *R. parkeri* is susceptible to ubiquitylation and subsequent elimination by Atg5 (autophagy-related gene 5)-dependent autophagy in mouse bone-marrow-derived macrophages. Both mutants were defective in spreading and causing diseases in mouse infection models for *R. parkeri* [100,131]. While the exact molecular mechanisms by which Sca5 prevents ubiquitylation of other surface molecules remain unresolved, the absence of Sca5 may have caused alterations to S-layers and exposed other susceptible residues in surface proteins [100]. Similarly, *R. parkeri* mutants lacking the expression of *wecA* and *rmlD*, two genes involved in the biosynthesis of O-Ag, are susceptible to ubiquitylation, suggesting that O-Ag polysaccharides modulate the surface protein assembly and prevent ubiquitylation [131,132]. Homologous *pkmt* genes have been identified in most *Rickettsia* species, but low pathogenic *Rickettsia* species often contain a frameshift mutation within the *pkmt2* gene, producing inactive PKMT2 [133]. Nevertheless, previous work determined that *R. typhi* and *R. australis* undergo ubiquitination and induce Atg5-dependent autophagy for pathogenesis [134,135]. It remains unclear whether PKMT1 is inactive in *R. typhi* and *R. australis*. Other unknown mechanisms may also contribute to the immune subversion of these two pathogens exploiting autophagosomes. However, the interplay of autophagy with *Rickettsia* during infections and its impact on pathogenesis remains complex and not well understood.

#### Actin-based motility and rickettsial spread

Several intracellular bacterial pathogens, such as *Listeria monocytogenes* and *Shigella flexneri*, assemble actin filaments on their surface and propel themselves using actin-based motility (ABM) to avoid host immune detection and invade neighbouring cells [136,137]. Besides arthropod vectors infected by different *Rickettsia* groups, the ABM is described as one of the major differences between TG and SFG rickettsiae [138,139]. Two actin-polymerizing determinants, RickA and Sca2, have been characterized for ABM in *Rickettsia*. The *rickA* and *sca2* genes are highly conserved in most SFG *Rickettsia* but missing in *R. prowazekii* [59,140,141]. Another TG agent, *R. typhi*, lacks *rickA* and expresses a divergent Sca2, forming short actin tails [60]. Unlike other bacterial pathogens, *Rickettsia* undergoes two different ABM phases regulated by unknown molecular mechanisms that alter the polar localization of each protein on the bacterial surface [142]. By comparing genome sequences of virulent and avirulent strains of *R. rickettsii*, a recent study identified RoaM (regulator of actin-based motility) as a cytosolic factor that mediates the ABM in *Rickettsia* [143]. However, it is unclear how RoaM interacts with other rickettsial determinants and modulates their functions in the ABM of *Rickettsia*. Once *Rickettsia* exits the endocytic vacuoles, the pathogen forms short actin tails with slow and curved motility using RickA, which is comprised of three domains: an uncharacterized *N*-terminal domain, a central proline-rich region with variable repeats, and a C-terminal Wiskott – Aldrich Syndrome (WASP)-like domain [144]. Bioinformatics analysis failed to identify signal sequences and transmembrane domains. Thus, it remains unknown how RickA is translocated to and remains on the rickettsial surface. Biochemical actin polymerization assays determined that the C-terminal WASP-like domain is sufficient to activate the actin-related proteins-2/3 (Arp2/3) heterodimer complex, a eukaryotic actin nucleator that organizes branched actin filaments [141,144]. During *R. parkeri* infections of endothelial cells, *R. parkeri* utilized RickA and exhibited ABM in curved trajectories. On the other hand, RickA expression was insufficient to promote ABM for *R. helvetica* and *R. raoultii* [145,146]. In *R. peacockii*, a non-pathogenic member of the SFG, a rickettsial insertion sequence (IS) element, ISRpe1, disrupted several genes, including *rickA*, potentially contributing to its low virulence [147]. Thus, the virulence functions of RickA for spotted fever rickettsioses remain unclear and require additional studies.

Following a replication phase with infrequent actin filament formation, SFG *Rickettsia* forms Arp2/3-independent long actin tails that provide fast motility and straight trajectories for intra- and inter-cellular spreads. Comparative genetic and insertional mutational analyses determined that Sca2 is responsible for the late-stage ABM of SFG *Rickettsia*. Sca2 is a rickettsial formin-like protein secreted via Sec-T5SS and harbours multiple noncanonical WASP-homology 2 (WH2) domains connected by folded structures of 45–55 amino acids in the passenger domain [110,148]. The WH2 domains are flanked by two proline-rich domains predicted to interact with the actin monomer binding protein called profilin. Interestingly, the *N*- and C-terminal repeat regions of Sca2 mediate intramolecular interactions and are predicted to form a circular shape to recruit two actin subunits for nucleation [148]. Similar to eukaryotic formins, recombinant Sca2 passenger domain interacts with the fast-growing barbed ends of actin filaments and competes with capping protein to facilitate dose-dependent actin nucleation activity in the presence of profilin. Thus, Sca2 displays formin-like activities on the rickettsial surface and contributes to the ABM of SFG *Rickettsia*. *R. rickettsii* and *R. parkeri sca2* transposon insertional mutants successfully evaded autophagic processes and displayed no growth defects in tissue culture cells. However, the mutants formed small plaques with minimal spread to adjacent cells and exhibited attenuated virulence in the animal infection models of spotted fever, suggesting that Sca2 is a virulence factor for *Rickettsia* [110,111]. In addition, *R. helvetica*, a member of the SFG *Rickettsia* predicted to be responsible for mild cases of spotted fever rickettsioses, possesses a full-length *rickA*, but has multiple premature stop codons in *sca2* and exhibited no ABM and reduced cytopathogenesis, further emphasizing the role of Sca2 in spotted fever pathogenesis [146].

While studies described above illustrate the biological significance of the ABM in rickettsial spread and pathogenesis, a recent study characterized another novel molecular mechanism that allows *Rickettsia* to spread to adjacent cells. Bioinformatic analysis identified that Sca4 is conserved in *Rickettsia* and contains two vinculin binding sites that share sequence homologies to mammalian talin-1 and *S. flexneri* IpaA2 [149]. This molecular mimicry enabled *R. rickettsii* Sca4 to interact with and activate vinculin, as evidenced by co-sedimentation studies and co-crystal structures of Sca4 with vinculin [149]. The Sca4 polypeptide sequence lacks the *N*-terminal secretion signal, but the C-terminal end of the protein was necessary for Sca4 secretion into host cells, suggesting that Sca4 may travel through T1SS or T4SS [150]. Microscopic images of cells infected with *R. parkeri* revealed that, unlike *L. monocytogenes* utilizing the ActA-mediated ABM for spreading, most *R. parkeri* lacks actin filaments in vacuoles protruding into neighbouring cells [150]. However, no significant differences were observed in the ABM between the *R. parkeri* wild-type and *sca4* mutant [150]. Instead, Sca4 is secreted into the host cytosol, inhibits the interaction between vinculin and α-catenin at the focal adhesion sites, and disturbs adherent junction complexes to reduce intercellular tension for efficient *R. parkeri* spread [150]. Thus, *R. parkeri sca4* transposon insertional mutant did not display abnormal attachment, invasion, or growth but formed small plaques in multiple cell types [150]. Recent studies provide significant insights into the complex molecular interactions between rickettsial effector proteins and host target molecules involved in the cell-to-cell spread. As orchestrated exit and spread is an essential step for successful rickettsial survival and pathogenesis, it is plausible that additional factors are involved in each stage of rickettsial spread. Thus, continued genetic analysis of *Rickettsia* is critical to uncover novel rickettsial factors and their interactions with host target molecules to reveal underlying mechanisms that regulate rickettsial pathogenesis.

## Undetermined roles of Rickettsial factors in arthropod colonization and transmission

The transmission of *Rickettsia* is mediated by various arthropod vectors (e.g. ticks, fleas, and lice). For successful disease transmission, pathogenic *Rickettsia* species must have evolved to adapt and survive in corresponding vectors, avoid immune surveillance systems, persist in key organ tissues, and utilize arthropod-derived molecules for survival strategies in diverse environments. However, the factors governing the susceptibility of any given arthropod species to *Rickettsia* infections are not well-defined. Comparative transcriptomic analysis of *R. conorii* infecting endothelial cells (HMEC) and AAE2 tick cells derived from *Amblyomma americanum* (lone star tick) strongly suggest that *Rickettsia* actively regulates different sets of genes during the infection of endothelium and tick cells [151]. Further, *R. rickettsii* in *A. aureolatum* (yellow dog tick) exposed to a temperature shift or blood meal acquisition altered global gene expression profiles, including genes involved in T4SS [152]. Similar observations have been documented for *Borrelia burgdorferi*, the causative spirochaetal agent of Lyme disease. Previous work extensively demonstrated that *B. burgdorferi* differentially regulates and expresses unique sets of genes for their successful infections of tick vectors and mammalian hosts [153]. Thus, it is highly probable that *Rickettsia* secretes novel virulence determinants enabling their survival and persistent transmissions within arthropod vectors to increase their fitnesses to arthropod vector species. However, we have limited understanding of the biological roles of rickettsial determinants involved in the vertical (transovarial transmission: from adult female to offspring and transstadial transmission: from immature stages to subsequent growth stages) and horizontal transmissions (acquired during blood-feeding) between the arthropod vectors and mammalian hosts.

Molecular epidemiological studies determined that the infection rates of SFG *Rickettsia* species depend on the infecting *Rickettsia* organisms and target tick species. Recent tick surveillance studies documented that the number of ticks infected with highly virulent *R. rickettsii* is much lower (<1%) than those infected with *Rickettsia* species of mild to low virulence, such as *R. parkeri* or *R. amblyommatis* [154–156]. Previous studies with artificial and *in vivo* tick feeding systems demonstrate that multiple factors, such as tick infection methods, *Rickettsia* and tick species, infection doses, and tick growth stages, contribute to the variability in rickettsial maintenance and transmission rates. For instance, *A. aureolatum* infected with *R. rickettsii* via animal feeding maintained the pathogen for multiple growth stages (larva, nymph, and adult) and generations [157]. Interestingly, immature ticks did not suffer from *R. rickettsii* infections, but the infected ticks exhibited a reduced oviposition success rate [157]. On the other hand, *A. cajennense* (Cayenne tick) failed to support stable *R. rickettsii* infections with inefficient vertical transmission rates [158]. In another experiment, laboratory rearing of field-collected *A. maculatum* (Gulf Coast tick) determined that *R. parkeri* infections do not reduce tick reproduction or survival [159]. By measuring multiple metrics, such as engorgement weight, nutrient conversion, egg production, and offspring viability, a recent study also determined that *D. variabilis* (American dog tick) and *A. maculatum* exhibit variable fitnesses and transmission rates for *R. parkeri*, *R. amblyommatis*, *R. rickettsii*, and *R. montanaensis* [160]. Based on these studies, it is posited that SFG *Rickettsia* species with low virulence are best suited to colonize ticks by vertical transmission routes, whereas highly virulent SFG *Rickettsia* species rely on horizontal transmissions to overcome negative impacts on tick viability and reproduction.

A recent study examined ticks-to-host transmission dynamics by allowing *R. rickettsii*-infected *D. variabilis* ticks to feed on naïve guinea pigs [161]. By PCR-detecting the presence of *R. rickettsii* at the bite site and distant tissue samples, this study determined that *R. rickettsii* transmission occurs as early as 30 minutes after tick biting [161]. As expected, the abundance of *R. rickettsii* increased as ticks continued to feed on guinea pigs [161]. However, it remains to be examined whether this rapid and time-dependent rickettsial transmission contributes to the disease severity in human patients. It has been posited that pathogenic *Rickettsia* species, including *R. rickettsii*, but not non-pathogenic *Rickettsia* species, can infect salivary glands, which permits their fast transmission along with immunosuppressive salivary molecules. For instance, *R. peacockii* and *R. buchneri*, non-pathogenic and endosymbiotic SFG *Rickettsia*, are known to infect ovarian tissues with limited tissue distribution in ticks [162]. However, a recent study demonstrated that *R. buchneri* is also present in the salivary glands of *I. scapularis* ticks [163]. Furthermore, comparative genome analyses identified multiple mutations within the coding sequences of putative virulence determinants, including RickA, Sca0, and Sca1 in *R. peacockii* and Sca2 and RickA in *R. buchneri* [164,165]. However, the absence of Sca2 or RickA did not alter the ability of *R. parkeri* to infect the midgut, salivary glands, and ovaries of *A. maculatum*, suggesting that Sca2 and RickA are dispensable for *R. parkeri* infection of tick organ tissues [166].

*R.felis* belongs to the TRG *Rickettsia* and causes emerging flea-borne rickettsioses worldwide as the primary vector (*Ctenocephalides felis*) exhibits a broad spectrum of host range and natural habitats. While the vertical transmission of *R. felis* has been described, the efficiency and fitness costs have not been carefully examined [167,168]. Interestingly, *R. felis* has been identified in the salivary glands of cat fleas, inducing changes in global transcriptomic profiles of infected fleas [169,170]. The horizontal transmission of *R. felis* has also been described in laboratory-reared cat fleas fed on an artificial feeding system [168]. Further, field studies determined the presence of *R. felis* DNA or *R. felis*-specific antibodies in small animals, suggesting their contributions to the horizontal transmission of *R. felis* [171–175]. A recent study corroborates these findings by demonstrating that *R. felis*-infected dogs became rickettsiaemic and allowed uninfected cat fleas to become infected with *R. felis* [176]. These studies suggest that domestic dogs, and other small animals, can serve as mammalian reservoirs for *R. felis* and contribute to the horizontal transmission of *R. felis* [176]. Genome sequences of *R. felis* revealed the presence of putative virulence factors (e.g. Sca proteins, phospholipases, ankyrin repeat proteins, tetratricopeptide repeat proteins) that are uniquely associated with the pathogen, along with multiple copies of *spoT* and toxin-antitoxin systems [75]. However, it remains unknown how these factors play out during the colonization of the cat fleas and transmission between the cat fleas and mammalian hosts.

The human body louse (*Pediculus humanus humanus*) serves as a primary vector for *R. prowazekii* [13]. However, unlike ticks and fleas, the body louse fails to be a reservoir for *R. prowazekii* because the infection leads to the death of the body louse. Instead, humans and eastern flying squirrel, *Glaucomys volans volans*, in the United States, can be persistently infected with *R. prowazekii* [177,178]. During the *R. prowazekii* infection of the body louse, the pathogen targets the epithelial lining of the midgut, leading to severe disruption of the digestive tract, releasing the *R. prowazekii*-infected blood into the haemocoel (making the body louse turn to red) [9,179]. The body louse excretes *R. prowazekii* with the faecal contents as early as three days post-infection [9]. *R. prowazekii* in the faeces is stable and remains infectious for several months; however, factors enabling this phenotype remain unknown [179]. Thus, the transmission of epidemic typhus occurs through the contamination of the bite site or mucous membranes with louse faeces contaminated with *R. prowazekii*. An experimental body louse infection model largely corroborated the earlier findings and characterized the kinetics and outcomes of *R. prowazekii* infections in the body louse [180]. Future studies with the body louse infection model may allow investigators to identify novel determinants contributing to the *R. prowazekii* infection of the epithelial cells of the body louse and *R. prowazekii* transmission to mammalian hosts.

## Concluding remarks

*Rickettsia* causes arthropod-transmitted febrile illnesses with divergent clinical symptoms ranging from self-limited mild conditions to severe life-threatening diseases with debilitating consequences. *Rickettsia* continues to evolve by removing nonessential genes for survival and replication and adapting to diverse environmental conditions. With recent developments in genetic tools and genome sequencing, investigations have identified novel *Rickettsia* species and characterized associated arthropod vectors. Further, comparative genome sequence analyses and mutational studies have suggested the presence of core genes and virulence determinants that may exhibit essential functions in rickettsial pathogenesis and colonization of target vector species. Recent studies highlight the complex molecular interactions between multiple rickettsial effector proteins and host target molecules. On the other hand, genome sequencing determined the presence of numerous hypothetical genes with no significant homologies to other known proteins, suggesting that *Rickettsia* provides a unique platform to reveal previously uncharacterized molecular mechanisms for obligate intracellular pathogens. Therefore, continued investigation of the bacterial effectors and host targets that mediate rickettsial intracellular lifecycle in mammalian hosts and arthropod vectors will enhance our understanding of this crucial mechanism of virulence and reveal cellular pathways that are exploited by pathogens during infection.
